# Essays and Lectures on Medical Subjects

**Published:** 1835

**Authors:** 


### Art. VIII.—Medical Experience.

*Essays and Lectures on Medical subjects by John P. Harrison, M. D. Professor of Materia Medica in Cincinnati College—Philadelphia—J. Crissey* 4 *Minor-street*, 1835.

This small work, seems to be especially dedicated to students of medicine; and unquestionably the subjects discussed in it, from their own intrinsic importance, well merit the serious consideration of those, for whom the book is designed. Yet, we would by no means be disposed to limit its circulation to students of medicine alone; for there is much contained in it, worthy the attention of the older members of the profession. The essay which first claims our notice, not only from its locality, but from the importance which the author himself seems to have attached to the subject, is on *medical experience* and occupies about one third the entire book. In this essay the author contemplates medicine in two aspects, as an art and as a science. He speaks of fallacies peculiar to each, but indicates strongly that the fallacy of "too much learning" is much the most dangerous, from it being often times shrouded in the splendors of "literary accomplishment." We know the proneness of human nature to love the mysterious, and to be captivated by the wonderful, and we feel assured that it is upon this wonder-loving principle with which humanity is so largely imbued, that the empiric in medicine is indebted for his success.

Although this is avowedly the age of enlightenment—although the progress of human knowledge has been such as to astonish even those who have kept pace with its improvement,—notwithstanding the arts and sciences have attained a degree of perfection, of which, the bare prediction but a few years ago, would have subjected the unhappy individual to the charge of lunacy; and our beautiful science, too ahcieving triumph after triumph, yet all have been unsuccessful in liberating the mind from the shackles of a delusion that has entwined it for centuries. Our author says:

> "Though delusion after delusion be exposed and perish, like images traced in sand by the sea-shore, which the next wave obliterates, yet the spirit of delusion, the love of deception, perish not, become not weary with the progress of years, with the experience of time. One form of error rises, is received with favor, eulogized as the messenger of great good to man, endures for a season, till another one arises to supplant its older rival, and like it, flourish in brief existence, then fades away and is forgotten.
>
> "The science, as well as art of medicine, to the eye of the philosophical beholder, resembles Virgil's mystical tree at the mouth of Avernus, thickly covered with visionary leaves, and on every leaf a dream. But it is a great and useful art, a noble and elevated science, well deserving of the highest appreciation, and most exalted esteem. For however obscured by error, and perverted by quackery, the art of healing is the source of many blessings to man. And as a science, medicine is of liberal bearing, and full of intellectual greatness; having for its object the expansion of the human mind on some of the most interesting topics that can invite investigation. Man, in his physical frame, in the organic structure, and physiological laws of that frame,—in his capacities of thought and emotion, and the inscrutable connexion of these divine capacities with his physical frame,—in the diseases so various and numerous, which afflict his body and mind,—the remedies which a sound experience has authenticated as most proper to remove those diseases,—and the collateral and auxiliary sciences, which pour in their contributions of aid, of light of power, into the storehouse of this great and noble science.—All these attest the dignity, and effectively proclaim the high and useful bearing of medicine on the well-being of mankind."

We do not claim for the author exclusive originality; but we say, he has set forth his views in an intrepid and manly way, and the general sentiment which pervades the essay, is such as to do honor alike to his head and heart. We are by no means either disposed to say that error does not exist, since this is incident to every human performance, and honesty obliges us whilst we award the meed of praise to that which is excellent in its kind, not to neglect or pass over that which may be error, and to our mind is error.

The author has remarked that language is the "interpreter of thought," and laments it as most unfortunate that it has been so frequently perverted from this its high function.—Indeed he remarks it is even more than the interpreter of thought. "It is embodied mind; it is tangible ideas; it is intellect made visible." Here we apprehend, we might with propriety take up the lamentation of the author and ask that all "ambiguity and imprecision be banished from the language of every cultivated man, who wishes to impart his thoughts with force, perspicuity and certainty." See page 13.

Medicine he contemplates as a physical science, and justly remarks, that it owes many of its proudest triumphs to the inductive philosophy—skill in the application of remedies, unwearied observation of the phenomena of their operation, and correct deduction, are those means which have tended most to the advancement of our science. It is a point conceded, that no science can be correctly taught or its principles explained, if the language used for such teaching or explanation, be ambiguous or indefinite. Hence our author has properly said, that "the terms of medicine should be well defined;" the language of physicians so definite as to convey unerringly "the precise ideas existing in their minds," and the nomenclature of our science divested of all ambiguity of expression, so that the human mind may be no longer retarded in its efforts after new discoveries, with which to adorn and ennoble our science.

"The science of medicine owes much to experience, but it is an experience at once discriminative and profound. The collected light of ages beams around her path. To the assiduous and enlightened cultivators of our art from the days of Hippocrates to the present age of the world, we are indebted for that fund of facts and rational deductions which go to constitute the science of curing diseases. No one individual, in this age, should arrogate to himself an original experience completely independent of the past experience of the medical world. Indeed, the most skilful in our art pretend to nothing more than a judicious combination of the experience of the past with their own observations—an application of that knowledge derived from erudition with that acquired by personal inspection, and a careful analysis of the opinions of their predecessors, so as to build upon it a manly and self-sustained judgment, which shall enable them to disentangle the perplexities of disease, and to send a discriminating eye over all those details of medical practice which are so apt to embarrass and confuse weak minds.

"Each physician must go through a discipline in the school of knowledge; each one has to advance from ignorance to wisdom by slow and measured steps; no man can, per saltum, reach the pinnacle of medical science; he might as well attempt to construct a dome without a foundation. All valuable acquisition is the fruit of toil."

In the further prosecution of this subject the author attempts to show "the sources of false medical experience, and in the next place proceeds to notice the best method of acquiring a true medical experience."

I. *False medical experience* he subdivides as follows: 1. Superstition, 2. Credulity, 3. Fallacy of testimony, 4. Ambiguity of language, 5. False theory.

It is not our purpose to go into a minute analysis of the author's remarks, upon each of these separate subdivisions; but simply to extract from each that which will convey a general impression of the whole.

1. *Superstition.*

"The three most intense of human predilections, considering man as an intellectual, social, and physical being, are the main avenues through which tyranny has made its most successful approaches, in rude states of society. These are, the strong desire man feels for the preservation of life, the irrepressible aspirings of his nature towards religious emotion, and his earnest want for some kind of polity. In ignorant and rude conditions of society, there is often a union of the functions of the physician, priest, and chief, in one individual. We need not, however, draw our illustrations in the present instance from barbarous tribes, for the celebrated Prince Hohenlohe, perhaps still alive, will furnish a most apposite example of such a species of folly.

"Alexander Leopold Hohenlohe, a Hungarian prince, whose father was disqualified for government by mental derangement, and whose mother was of an ardent temperament, and of fervid zeal for her religion, began in 1815 to manifest his talents for preaching agreeably to his fond mother's hopes and entreaties. 'After having received the papal permission to consecrate three thousand rosaries, crucifixes, &c. at once, he left Rome and went to Germany, where he was considered by his colleagues as devoted to Jesuitism, and an enemy to knowledge.' Martin Michel, in Baden, a worker of miraculous cures, through the power of faith, and the efficacy of prayer, assured the prince, that the faith and prayers of a prince, were of much greater prevalence and potency to effect miraculous cures than were those of a common peasant, such as Michel was. The imagination of the fanatic prince was fired at once, and he soon proceeded to put into execution Michel's plan of healing the halt, the blind, the impotent, and the insane. A princess, Matilda, of Schwartzenburg, who had distortion of the spine, was made to walk through the agency of the princely priest and doctor, aided by Michel, his guide, encourager, and friend. The prince soon, however, commenced operations on his own responsibility and prayers, and multitudes eagerly crowded around his path. This spiritual physician's fame soon diffused itself over Germany, the country of the ideal, and reached even England and America. His enthusiastic admirers published many, in their opinion at least, veritable cases of terrible disease, which they solemnly averred were cured by the great apostle of delusion. He could not succeed in his cases in the hospitals of Wurtzburg and Bamberg, and a prince of Hiedburghaosen, who labored under an affection of the eyes, in consequence of discontinuing all medical applications, and relying on this spiritual doctor, found his eyes soon get much worse. Still the confidence of Hohenlohe was so great, and his presumption so overweening, that in 1821, he solicited the Pope's attention to his miraculous endowment, but Pius VII. intimated his will to be, that the process should not be designated a miracle, but *priestly prayer for healing.*

"After Hohenlohe's failure upon the prince, with diseased eyes, he declared himself exhausted, and was unwilling to be seen by the health police whilst in the act of performing his cures. Of late, he works at a distance, and the newspapers, and some of the medical periodicals, have given us accounts of the reputed miracles performed by his highness, or excellency, in France, England, Scotland, Ireland, and the United States. His mode of late, is to direct the afflicted person, making application for cure to him, to pray at certain times of the day, cotemporaneously with himself. It has been objected to these simultaneous prayers by some caviling spirits, that a prayer at 8 o'clock, in Hungary, has been ended before that of 8 o'clock, at Marseilles, begins; but to this objection, urged by the sceptic, one irrefutable reply is enough, it is all miracle, or all superstition—from alpha to omega—and admits no investigation, but demands a simple, unquestioning faith, too facile for doubt, and too submissive for reply.*

"The above furnishes a melancholy proof of the perverted application of that scheme of human illumination, which Christianity furnishes, and is a signal verification of a remark already made, that mankind love the luxury of delusion, and seek its gratification in the most exorbitant exhibitions of folly."

### 2. *Credulity.*

This vice, the author contemplates as near akin to superstition; and certainly, since they both emanate from one prolific parent, *ignorance*, their relationship will not be doubted. When this passion predominates in the human mind, there is no proposition, either in Christian ethics or in medicine, too absurd or preposterous not to receive full credence. It is not asked that it should be sustained by facts—that experience and observation should be the touchstone by which its truths might be demonstrated. The author proceeds to say:

* Encyclop. Americana: article, Hohenlohe.

"A warm and animated zeal for any cause, unless that zeal is chastened and regulated by a severe discipline of judgment, is sure to mislead the mind. Quod volumus, facile credimus, is as true in medicine as in the ordinary pursuits of life. Our science is filled with convincing illustrations of this position. The discoverer of every new remedy asserts that he finds in it an epitome of the whole *materia medica.* The metallic tractors of Perkins, the animal magnetism of Mesmer, the elixir of life of Paracelsus, the alkahest of Van Helmont, and the panaceas and catholicons of the present day, all have derived their source from fraud operating on credulity. Even where no sordid considerations influence the propagator of a revived, or newly discovered remedy, the ardent enthusiasm of the experimenter urges him to great extravagance in the commendations he bestows on it. Baron Storck greatly overrated the remedial power of the Narcotics, upon which he made so many experiments. There is no prominent remedy in our catalogue of agents for the removal of disease, but what has been at one time surrounded by a mist of panegyric. And how many inert substances have been eulogized, that are now quietly slumbering in the tomb of forgetfulness! Opium, digitalis, mercury, antimony, and the bark are the most effective weapons of medicine. Each of these have been injured in their legitimate character, as medicinal substances, by the indiscriminate encomiums lavished upon them. The most enlightened physicians now agree with the illustrious Sydenham, 'that the practice of physic chiefly consists in being able to discover the true curative indications, and not medicines to answer them; and those that have overlooked this point have taught empirics to imitate physicians.'"

Again our author remarks:

"Under the head of credulity, most appropriately, perhaps, are to be included the reveries and figments of Hahnemann's subtle but prolific brain. Endowed to a degree far beyond the common rate of even Germanic ideality and metaphysical vapouring, the author of the system of Homæopathy, has challenged human credulity to the utmost, and put to test the strength of man's addictedness to abandon his mind to the wildest wanderings of hypothesis: if the hypothesis is enforced by confident appeals to testimony, and a determined hostility towards all established views. Mixing up with the crudest modes of explaining the action of remedies, much parade of learning and professions of philanthropy, the abettors of this system, most emphatically denounce and villify all the generally established modes of considering, and of treating, disease. The captivating blandishments of infallibility and impeccability are, of course, the supreme features of homæopathism. It is the wisest, safest, best, and most sure mode of treating all human maladies. All other plans of conducting the treatment of disease are, in comparison to the homæopathic, but mere clumsy artifices of medical skill, mere potent engines of perpetuating morbid action, and of perpetrating death. Hahnemann, like another Bacon, has promulgated his organon, the leading doctrinal peculiarity of which is, *similia, similibus curantur*, in accordance with which to cure a disease, you must administer medicines that will excite symptoms similar to the disease—and thence is derived the name of the system, homæopathy, or as it is sometimes written, homoopathy—from the Greek words, *omoion* alike, and *pathos* disease."

### 3. *Fallacy of Testimony.*

In the long list of agents, which have exerted a potent influence in retarding the advancement of medical science, probably none outranks the fallacy of medical testimony.—False facts have been the bane of medical improvement, and as the author has truly remarked, the materia medica has in an especial manner been subjected to the ruinous and blighting effects of false experience. The cause of this however is obvious. Intimately associated with the remedy is the disease for which it is prescribed—hence the deductions of cure from the application or use of a particular plant, have been made upon insufficient data, and without that mature reflection and deliberation, which should at all times characterise the speculations of the philosophic and intelligent physician; or it has been done for the sordid purpose of making money, even at the expense of human life.

In illustration of this division of the subject the author instances among many others the case of Stone, said to have been cured by the celebrated Mrs. Stephens, whom the British Parliament so liberally compensated, after the individual was pronounced cured by several of the most enlightened medical men of that day. But this man was not cured—the stone could not be detected by the sound, because it was lodged in a pocket formed probably by a fold of the bladder. An interesting case of this kind, is reported by Professor Jameson in the present number of the Journal; in which by the most patient and laborious investigation and research, he was for a long time unable to detect the calculus, and yet the man had every symptom of the disease. The pocket in which this lodgment of calculi took place, was not discovered till the finger was introduced into the bladder, through the opening made for the extraction of the stone. See page 216.

"It were a task of immense labor to advert, by special enumeration, to all the numerous cases of such fallacy, as that arising from extravagant views of the medical properties of the various agents, which from age to age, and from year to year have received the unsparing and undistinguishing eulogies of physicians. And not contented to expatiate on the virtues of medicines which the ingenuous dealer in drugs kept, appropriately labeled, in his shop, the most accomplished physicians have at times departed from the employment of the remedies known to the profession, or recommended by men of scientific candor, and too enthusiastically attached themselves to the use, and too eagerly recommended remedial agents, whose composition was a secret, and the authors and vendors of which, knew nothing of the just principles of medical science.

"The splendor which at one time surrounded the reputation of Swaim's Panacea, was derived from the dazzling brilliancy of that light which several of the luminaries of our American medicine threw around it. With precipitate and onward haste, some of our most enlightened physicians were found swelling the loud chorus of praise which was sounding forth the many virtues and transcendant excellencies of this nostrum. Certificates, signed by several of the most eminent medical men of the country, quickly found their way into the newspapers, and were by them rapidly disseminated through the Union."

Here we have an objection to the manner in which the author has attempted to gloss over the conduct of "certain luminaries of American medicine" (as he terms them) in certifying to the virtues and efficacy of Swaim's Panacea. Such conduct would be reprehensible in the humbler members of the profession; but when men occupying the most dignified and elevated places in medicine, prostrate the nobler purposes of their profession, to pander to the vile cravings of a sordid *empiric*, the scorpion lash of an indignant and dishonored profession should be exerted to correct the abuse and not tamely rest satisfied with their simple acknowledgement that they had "overrated the value of the Panacea of Swaim, and have for a long time ceased to prescribe it." Why ever prescribe it? Were they not emphatically acting the part of the *charlatan* in prescribing a panacea the constituents of which they knew not? We should like indeed to see the difference pointed out between the *nostrum vender* and these *certifiers* to its virtues. If there is any, the advantage is on the side of Swaim, who knows nothing, neither pretends to know any thing, of the exalted principles of medical science; whilst these, are proud and distinguished teachers in medicine.

We would here in conclusion express the hope that they have read with careful attention the report of the Medical Society of Philadelphia, drawn up by Horner, Harris, Klapp Meigs and Bell, and have resolved for the future, that they will not encourage the dissemination of nostrums by the influence of their names even though great *pecuniary loss* should be sustained.

4. *Ambiguity of Language.*

We agree with the general tenor of the author's remarks under this head and assume the position as incontrovertible that accuracy of language is the only sure method of making ourselves perfectly intelligible to those whom we are addressing

5. *False Theory.*

It does seem to us that the author under this head is liable to the charge of endeavoring to establish a theory if not false at least unsupported by that mass of testimony and well ascertained fact which are adduced to sustain the doctrine of the humoral pathology as taught and believed at the *present time.* He does not treat the doctrine with candor—he does not give it that consideration to which it is entitled—he speaks of the old exploded views of the humoral pathologists in reference to "lentor, alkalescency, fermentation and concoction and in the pride of opinion asks, "what has humoralism ever done for medicine?" This question he answers himself. "It has retarded its march, it has shut up the avenues to its successful cultivation, and deteriorated the genuine spirit of correct investigation."

We ask, is it fair to treat a grave doctrine by one fell swoop of denunciation? We apprehend not. If the author had cited the facts upon which the modern humoral pathologist rests the truth of his doctrine and refuted them by sound argument and correct logic, it would have been well; but he has repudiated them as the "chimeras dire" of a fruitful imagination. On page 47, he says:

"A boundless terra incognita, as I have stated in another place,* is presented by the mass of circulating vital fluid, contained in the arteries and veins of the animal machine, which our fond visionaries and remance writers on medicine have peopled with every variety of direful foes, to the weal of the body. Sometimes animalcula are made to sport in cruel triumph along the conduits of life; at other times, the blood is wrought into woful chemical changes, and is thickened into a dark spissitude or lentor, or thinned into a mere cruor, according as the mind of the medical novelist is troubled with 'thick coming fancies,' or is elated by etherial visions. One will gravely write by the hour, of impregnations, foreign infusions, and certain mysterious, but yet unappreciable

* Harrison on Cholera—Baltimore Medical and Surgical Journal; July, 1834.

changes in the vital fluid. Another will dwell with infinite complacency on the horrible blackness of the blood, which he finds dark as Cerbebus, and fit only for congestions, collapses, and death. But a still additional writer shall entertain his readers, very little at his own expense of research, by expatiating over the ground of an ill-defined and misty space, from which he imagines he descries in clear perspective, both territories of humoralism and solidism. Such a ready scribe speaks you fair, and tells you in grandiloquent phrase, of the possibilities, nay, absolute probabilities, of the blood becoming corrupted, and refers you for proof, not to the chemical analysis of any poisonous products found in the circulating mass, but to the experiments of some gentle vivisectors, who veritably force, by their torturing process, foreign materials into the blood vessels. Medicine is *called* by these gentlemen, a demonstrative science—aye, they emblazon it by the proud titles of an inductive and certain science,—a department of human knowledge, requiring at each step of its glorious march to ultimate perfection, a precision in the collocation of its facts, and a fixedness in its positions, that can never be impaired by the revolutions of time. Fond conceit! Is not instability written on the face of our science, and shall they who yet foster the prejudices of the past, in favor of a doctrine that never has been proved, idly dream of the ultimate achievements of truth over error, when they are engaged in rebuilding the crumbling fabric reared in the dark ages of human knowledge! We demand the proofs—we require, before we give our belief to the undigested doctrines of humoralism, to see something like a rigid and scientific appeal made to analysis and demonstration. Who ever appeals to the chemical changes of the elementary constituents of the blood, either during life, or after death, for evidences of the state of morbid action that may be present, or has left its ravages behind? It is evident that an incalculable amount of false experience in medicine, must have been reared upon the conceptions of disease, and the modus agendi of remedial agents, adopted by the defenders of the humoral doctrine;—such conceptions, in the language of Cullen, having contributed nothing to improve, and having often misled the practice of physic.

"Andral, who, to a very limited extent, is a humoralist, candidly avers, "that in the present state of the science, it is the part of a sensible man not to adopt the doctrine of humorism too lightly, by judging from facts, many of which require a re-examination before they are finally admitted, and that we ought to be particularly on our guard against being in too great a hurry to make practical applications of it.'*"

Here we conceive the author to be not so felicitous as usual in his reference to M. Andral; the support he expected his views of solidism to derive from this great anatomist and pathologist would have been swept from him had he even proceeded to finish the quotation begun, much less to refer to Andral's entire chapter on the lesions of the blood.

---

* Andral's Anatomy, vol. 1, p. 419.

It has been ascertained beyond contradiction that the blood does undergo extensive alterations; this has been established by actual experiment as well as by chemical analysis.

* M. Beclard cites a case in which he detected a solid clot of blood filling the heart and trunks of the large vessels, the interior of which presented a mass of encephaloid matter. Velpeau also mentions an instance of a mass of encephaloid concealed in a clot of blood filling the vena cava. In his *Anatomie generale*, Bichat reports a case in which the vena porta and the hepatic and splenic veins are filled even to their most minute extension with a greyish sanies instead of blood. The experiment has been instituted again and again of injecting the veins of one animal with the blood of another, both being in health and no injury resulting and yet when the blood from a diseased individual has been thrown into the veins of one in health consequences the most frightful have resulted.

M. Gendrin in his work on inflammations and in another on fevers reports many experiments made upon animals by injecting into their veins and cellular tissue, blood abstracted from patient's laboring under putrid fever and others afflicted with small pox. In these cases the most severe symptoms were speedily manifested and death soon ensued.

Now if experiments conducted in the true spirit of philosophic inquiry by different individuals at different times are to be relied on, do they not conclusively establish the position that the nature of the blood is altered, and if changed why will not a corresponding alteration be produced upon the solids? Can the solids exist without their regular and constant supply of blood? neither can the blood be formed without the intervention of the solids. Here then is the establishment of a beautiful reciprocity of action for the purpose of imparting life and animation to every tissue and organ such only as could have been effected by the great architect of nature.

"Thus then in the three-fold respect of the vital phenomenon, intimate structure, and chemical composition, "says Andral," no line of de-

* Andrel's Anatomy.

marcation can be drawn with strictness and precision between the blood and the solids. Physiologically speaking, it is impossible to conceive that one of these two parts of the same whole could be modified without the other being so likewise. On the one hand, inasmuch as the blood nourishes the solids, and as without its presence they cannot support life, the state of the solids cannot but be influenced by the state of the blood. The chemist might as well say that the nature of a body does not depend on the nature of the elements that compose it. On the other hand, the solids, considered with respect to their relations with the blood, form but two classes: the one contributing to *make* the blood, such as those concerned in the actions of absorption, digestion, arterial circulation, and respiration; the other contributing to *unmake* it, those, namely, concerned in the actions of venous circulation, secretion, and nutrition. No one solid, therefore, can undergo the slightest modification, without producing some derangement in the nature or quantity of the materials destined to form the blood, or to be separated from it. Physiology, then, leads us to the conclusion that every alteration of the solids must be succeeded by an alteration of the blood, just as every modification of the blood must be succeeded by a modification of the solids. Viewed in this light, there is no longer any meaning in the disputes between the solidists and the humorists; the system appears to constitute but one great whole, indivisible in the state of health as well as in that of disease; the division of the parts of the body into solids and fluids, seems to be a distinction of small importance, and one that is not always just, since it ceases to exist in the intimate structure of the organs, in which all the grand vital phenomena take place, and in which also, occur all the changes that constitute the morbid state.

"This intimate and necessary dependence of the blood and solids on each other having been once taught us by physiology, what remains to be done? Merely to have recourse to observation for facts, and to draw legitimate conclusions from them."

We have neither time nor liesure to pursue the investigation of this subject further at present neither will the space allotted to this review admit of its further extension.

*Method of acquiring true experience.*—It is here the author has shown himself master of his subject. The clear, beautiful, and interesting manner in which he has placed this subject before the mind of the learner will at once arrest his attention and exhibit to him the fact that it is only by patient industry and perseverance in the pursuit of medical knowledge that he can ever hope to arrive at distinction in his profession. The author happily remarks that

"To the formation of a sound discriminating judgment in practical medicine, there can be no substitute for clinical observation. Whatever attainments the student may make in anatomical, physiological, and pathological knowledge, still he must have his faculties exercised at the bedside, to enable him to acquire a just and useful experience in the art of curing disease. There can be no substitute for this most essential mode of gaining a knowledge of the symptoms and nature of the various maladies to which the human economy is subject. All other acquisitions are only preparatory to the acquirement of clinical experience. Without witnessing the diversified phases of disease, as they reveal themselves substantially and truly at the snffering patient's couch, all our attainments, however valuable as a preparation, are useless as an end. As a terminating point of practical utility, it is very patent to the most obtuse interest, that the most accurate views of the organization of man's composite frame, or of the laws of healthy action established in that organization, or even of the laws of morbid action, could never impart enlightened views of the symptomatology and therapeutics of disease. No one can be a scientific physician unless he is intimately acquainted with the anatomy and physiology of the body—but neither can he be a judicious and safe practitioner, unless he superadd to all such most necessary preparatory knowledge, the knowledge of disease as it is derivable from seeing it—and of realizing its presence and power, and many changes, by an actual examination of the suffering fellow being, whose system is prostrate under its powerful agency. We cannot insist too strenuously on this point—it is of vital consequence to the growth of a legitimate experience in medicine—and without it, without clinical observation—whatever embellishments collateral branches of knowledge may pour around our art—whatever most necessary investigations may be had into anatomy, physiology, and pathology, as these fundamental divisions of our science are learned in dissections, lectures and books, yet at last the student of medicine must acquire for himself, by an exploration of the phenomena of disease at the bedside, the crowning excellence of all useful experience."

The entire book is constituted of four remaining essays and lectures each worthy of a separate and distinct notice; but for the sake of brevity we will speak of them as a whole and recommend them to the attention and perusal of students of medicine and at the same time express the hope that our elder brethren of the profession will read them and profit much by the instruction they contain. C. R. C.

